# Correction of β-thalassemia mutant by base editor in human embryos

**DOI:** 10.1007/s13238-017-0475-6

**Published:** 2017-09-23

**Authors:** Puping Liang, Chenhui Ding, Hongwei Sun, Xiaowei Xie, Yanwen Xu, Xiya Zhang, Ying Sun, Yuanyan Xiong, Wenbin Ma, Yongxiang Liu, Yali Wang, Jianpei Fang, Dan Liu, Zhou Songyang, Canquan Zhou, Junjiu Huang

**Affiliations:** 1 Key Laboratory of Gene Engineering of the Ministry of Education, Guangzhou Key Laboratory of Healthy Aging Research and State Key Laboratory of Biocontrol, SYSU-BCM Joint Research Center, School of Life Sciences, Sun Yat-sen University, Guangzhou, 510275 China; 2 Key Laboratory of Reproductive Medicine of Guangdong Province, School of Life Sciences and the First Affiliated Hospital, Sun Yat-sen University, Guangzhou, 510275 China; 3 Department of Pediatrics, Second Affiliated Hospital, Sun Yat-sen University, Guangzhou, 510120 China; 4 Verna and Marrs Mclean Department of Biochemistry and Molecular Biology, Baylor College of Medicine, One Baylor Plaza, Houston, TX 77030 USA

**Keywords:** β-thalassemia, *HBB* −28 (A>G), base editor, human embryo

## Abstract

β-Thalassemia is a global health issue, caused by mutations in the *HBB* gene. Among these mutations, *HBB* −28 (A>G) mutations is one of the three most common mutations in China and Southeast Asia patients with β-thalassemia. Correcting this mutation in human embryos may prevent the disease being passed onto future generations and cure anemia. Here we report the first study using base editor (BE) system to correct disease mutant in human embryos. Firstly, we produced a 293T cell line with an exogenous *HBB* −28 (A>G) mutant fragment for gRNAs and targeting efficiency evaluation. Then we collected primary skin fibroblast cells from a β-thalassemia patient with HBB −28 (A>G) homozygous mutation. Data showed that base editor could precisely correct *HBB* −28 (A>G) mutation in the patient’s primary cells. To model homozygous mutation disease embryos, we constructed nuclear transfer embryos by fusing the lymphocyte or skin fibroblast cells with enucleated *in vitro* matured (IVM) oocytes. Notably, the gene correction efficiency was over 23.0% in these embryos by base editor. Although these embryos were still mosaic, the percentage of repaired blastomeres was over 20.0%. In addition, we found that base editor variants, with narrowed deamination window, could promote G-to-A conversion at *HBB* −28 site precisely in human embryos. Collectively, this study demonstrated the feasibility of curing genetic disease in human somatic cells and embryos by base editor system.

**Electronic supplementary material:**

The online version of this article (doi:10.1007/s13238-017-0475-6) contains supplementary material, which is available to authorized users.

## Introduction

The explosive growth of human genomic data has revealed unprecedented numbers of disease-causing point mutations. Repairing such mutations may offer the best, and in some cases, only cure for genetic diseases. We and other groups have sought to correct disease mutant by combining CRISPR/Cas9 and homology directed repair (HDR) in human tripronulcear zygotes and diploid zygotes. However, low efficiency, mosaicism, off-target cleavage, and unintended homologous recombination (between target site and endogenous homologous genomic DNA sequence) remain obstacles that hamper the clinical applications of such approaches (Kang et al., 2016; Liang et al., 2015; Tang et al., 2017). In a recent report, it was found that diploid human zygotes, distinct from pluripotent cells, tends to repair DNA double strand break (DSB) using endogenous homologous sequence (Ma et al., 2017), consistent with what we have found in human tripronuclear zygotes (Liang et al., 2015). In the study, highly efficient repair of the mutant allele was achieved using the wild-type (WT) allele in heterozygous human zygotes through CRISPR/Cas9 (Ma et al., 2017). However, homozygous mutant embryos could not be repaired in way because of the lack of WT alleles. Additionally, recombination may occur with similar but not identical endogenous sequences, leading to unexpected mutations, as we found in human tripronuclear zygotes in which *HBB* recombined with *HBD* (Liang et al., 2015). Using base editors to directly repair point mutations may represent an efficient and highly specific alternative.

The base editor is a RNA-protein complex, adapted from the CRISPR/Cas9 system and cytidine deaminase (Komor et al., 2016). The effector protein is composed of cytidine deaminase (rAPOBEC1), Cas9, and uracil DNA glycosylase inhibitor (UGI). It can deaminate cytidine (C) to uridine (U) without inducing DNA DSB, and finally result in C-to-T (or G-to-A) conversion in the target DNA sequence (Hohmann, 2017; Komor et al., 2016; Liang et al., 2015). Efficient base editing at single-base resolution has been reported in plant, yeast, human cells, mouse zygotes, and human tripronuclear zygotes (Chen et al., 2017; Kim et al., 2017b, c; Komor et al., 2016; Li et al., 2016, 2017a, b; Liang et al., 2017; Lu and Zhu, 2016; Ren et al., 2017; Zhou et al., 2017); Zong et al., 2017). Intriguingly, mouse embryos and pups with 100% point mutation efficiency (free of mosacism), as well as human tripronuclear zygotes has been generated (Kim et al., 2017c; Li et al., 2017a; Liang et al., 2017). However, whether base editors can repair homozygous T>C (or A>G) disease mutant in human embryos remains to be tested.

β-Thalassemia, a common genetic disease in Mediterranean countries, North Africa, the Middle East, India, Central Asia, and Southeast Asia, is a major problem of global health (Cao and Galanello, 2010; Galanello and Origa, 2010; Weatherall, 2010). Genetic mutations, which will lead to the reduction of hemoglobin β chain (β-globin) and erythrocytes, finally cause oxygen shortage, bone deformity, organ dysfunction and even organ failure in many parts of the human body (Cao and Galanello, 2010). Based on the severity of the disease, β-thalassemia can be classified into β-thalassemia minor (also called β-thalassemia carrier), β-thalassemia intermedia, and β-thalassemia major (Cooley’s anemia) (Cao and Galanello, 2010). Without treatment, patients with β-thalassemia major usually die before age 5. Thalassemia major patients require lifelong blood transfusion and iron chelation treatment to survive, often accompanied by numerous complications, including arrhythmia, congestive heart failure, hypothyroidism, hypoparathyroidism, hypogonadism, diabetes, osteoporosis, liver cirrhosis, and infection (Chern et al., 2007; Wu et al., 2017). To date, allogeneic bone marrow transplantation (BMT) is the only curative therapy, but BMT is limited by human leukocyte antigen (HLA) compatibility. β-Thalassemia is mainly caused by mutations in the *HBB* gene, of which −28 (A>G) mutation is a common defect reducing the transcription of *HBB* (Orkin et al., 1983). Patients with homozygous or compound heterozygous −28 (A>G) mutation may develop severe anemia or intermedia anemia (Cao and Galanello, 2010; Orkin et al., 1983). Correcting the −28 (A>G) mutation by base editing should help to ameliorate anemia. Here, we report the efficient correction of −28 (A>G) mutation in human primary cells and human embryos by base editors.

## Results

### Correcting *HBB* −28 (A>G) mutation in human cell line by base editor

Of the two base editors (BE), BE2 (rAPOBEC1-dCas9-UGI) and BE3 (rAPOBEC1-nCas9-UGI), BE3 showed higher editing efficiency (Kim et al., 2017a). We therefore decided to repair *HBB* −28 (A>G) mutation using BE3. *HBB* −28 (A>G) mutation, in which the wild-type A at position −28 (A_−28_) is replaced with G in patients (G_−28_), locates in the ATA box upstream of the first exon of HBB (Fig. 1A) (Orkin et al., 1983). Three gRNAs targeting this mutant *HBB* allele were designed to convert C (on the complementary strand) to T (Figs. 1A and S1). We found that G at position −25 (G_−25_) might also be converted to A by these gRNAs (Fig. 1A). To test the deamination activity of these three gRNAs, we cloned the DNA fragment surrounding the *HBB* −28 (A>G) mutation into a lentiviral vector for stable integration in 293T cells. After selection with puromycin, three different cell clones were picked and verified by PCR (Fig. 1B). PCR primers (FP1 & RP1), that could specifically amplify this exogenous *HBB* −28 (A>G) mutant fragment, were designed (Fig. 1B). Sanger sequencing of this PCR amplicons indicated a clear G at *HBB* position −28 in these cell clones (Fig. 1B).

**Figure 1:**
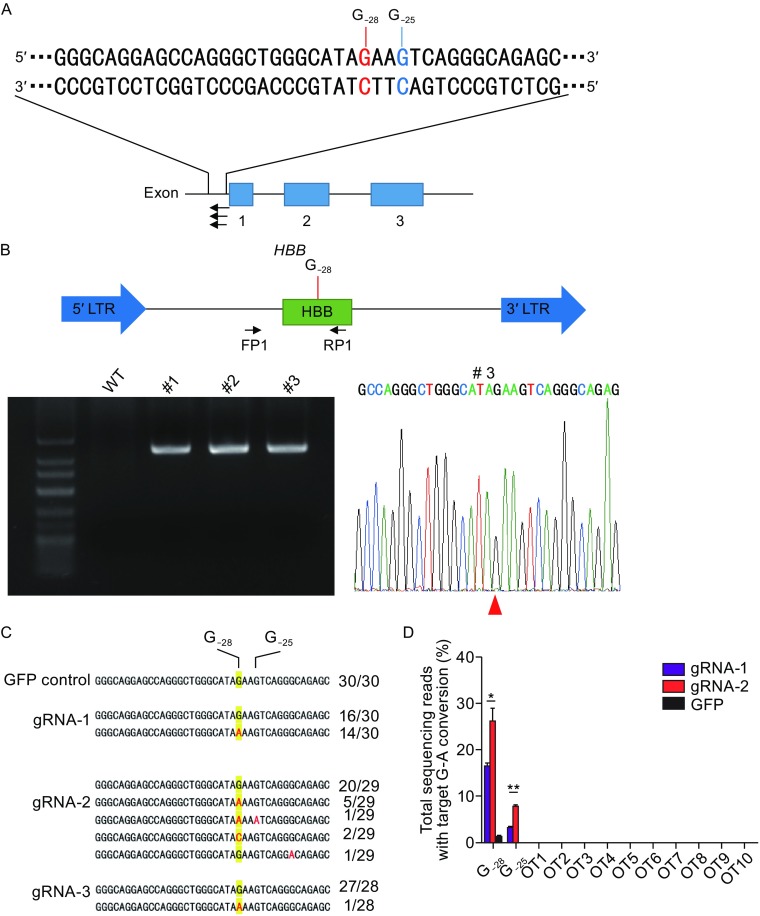
**Correcting** ***HBB*** **−28 (A>G) mutation in human cell line**. (A) Schematic of *HBB* −28 (A>G) mutation. The exons are labeled with blue boxes. −28 (A>G) mutation was in red and indicated with red line (G_−28_). The −25 (G), next to G_−28_, was in blue and indicated with blue line (G_−25_). And gRNAs were labeled with black arrow. (B) Generation of *HBB* −28 (A>G) mutant stable cell lines. A fragment of *HBB* gene, containing the −28 (A>G) mutation, was cloned into a lentiviral vector. Packaged lentivirus was used to infect 293T. Virus-infected cells were selected by puromycin. 7 days after selection, single clones of cells were picked. The up panel showed the design of the recombined lentivirus vector. *HBB* gene fragment containing −28 (A>G) mutation was labeled with green box. LTR (long terminal repeat) region of lentiviral vector was labeled with blue arrowhead. PCR primer used to specifically amplify *HBB* fragment from integrated provirus were showed. The down panel showed the results of one wild-type 293T cells and three clones, amplified using FP1 and RP1. Representative sequencing chromatographs of the PCR amplicons of #3 clone were shown. The mutant base (G_−28_) was indicated by red arrowheads. (C) Precise repairing of *HBB* −28 (A>G) mutation by base editor 3 in the *HBB* −28 (A>G) mutant stable cell line. TA cloning sequencing showed clear G>A conversion at the target site. The frequency of each allele is shown. (D) Deep sequencing to detect on-target and off-target deamination at 10 potential off-target sites in *HBB* −28 (A>G) mutant stable cell line. Bars represent mean ± SEM (*n* = 3). Significance was calculated using a two-tailed unpaired *t* test (*P < 0.05, **P < 0.01)

Next, we co-transfected the gRNA and the BE3 expression vectors into clone #3. Cells transfected with GFP were included as a control. After 48 h, the cells were harvested. Target sites were amplified with FP1 and RP1 primers. Sanger sequencing of the PCR amplicons revealed obvious G>A conversion using the three gRNAs (Fig. S2). TA cloning and sequencing further confirmed active conversion in these cells (Fig. 1C). The conversion efficiency was 46.7% (14/30) for gRNA-1 (Fig. 1C). And consistent with previous findings in human cells and mouse embryos, we found proximal-site deamination using gRNA-2 (Fig. 1C) (Liang et al., 2017). Off-target deamination could be a concern in base editing, so we further investigated off-target deamination in this *HBB* −28 (A>G) mutant cell line. We again co-transfected BE3 together with either gRNA-1 or gRNA-2 into clone #3. GFP transfected cells were used as a control. The cells were harvested for genomic DNA extraction 48 h after transfection. The exogenously integrated *HBB* DNA fragment and 10 potential off-target sites were PCR amplified for deep sequencing. We found 16.3% and 26.0% G>A conversions at the target sites for gRNA-1 and gRNA-2 respectively, significantly higher than the rate of 1.2% in GFP control cells (Fig. 1D). And in line with data in Fig. 1C, we found that both the G_−28_ and G_−25_ at the target region could be deaminated by BE3 (Fig. 1D). We found higher G>A conversion efficiency at G_−28_ and G_−25_ using gRNA-2 (Fig. 1D). Moreover, we did not found any off-target deamination at the 10 potential off-target sites examined for both gRNAs, indicating high specificity (Fig. 1D). Taken together, these results clearly indicate the feasibility of repairing *HBB* −28 (A>G) in human cells *in situ* by base editing.

### Correcting *HBB* −28 (A>G) mutation in primary skin fibroblast cells of a β-thalassemia patient by base editing

Inspired by the high efficiency and specificity of repairing *HBB* −28 (A>G) mutation by base editing, we sought to correct *HBB* −28 (A>G) mutation in patient’s cells. We isolated and cultured the skin fibroblast cells from a homozygous −28 (A>G) mutant patient (Fig. 2A and 2B). After transfection of BE3 and gRNA-1 into these cells by nucleofection, we achieved 80%–90% transfection efficiency (Fig. S3). At 48 h after transfection, the cells were used for single cell sorting (Fig. 2C). The sorted cells were whole genome amplified by multiplex displacement amplification (MDA), and then the *HBB* locus was PCR amplified (Fig. 2C). Here, we also observed efficient repairing of the homozygous mutation to heterozygotes or WT bases as shown by Sanger sequencing.

**Figure 2:**
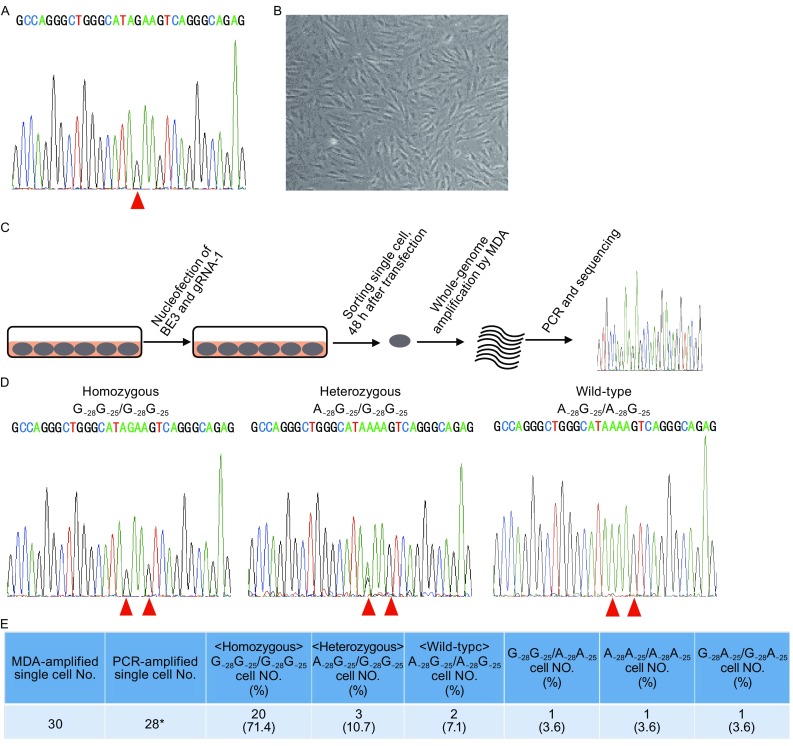
**Correcting** ***HBB*** **−28 (A>G) mutation in primary skin fibroblast cells of beta thalassemia patient**. (A) Sanger sequencing to detect the genotype of the patient. Genomic DNA from the patient’s cells was extracted for PCR amplification of the target region. PCR amplicons were then sequenced by Sanger sequencing. *HBB* −28 (A>G) mutation were labelled with red arrowhead. (B) Primary skin fibroblast cells from the *HBB* −28 (A>G) mutant patient. (C) Schematic of base editing in *HBB* −28 (A>G) homozygous mutant skin fibroblast cells and single cell genotyping. Skin fibroblast cells were transfected with BE3 and gRNA-1. 48 h after transfection, single cell was isolated and whole genome amplified. The genomic DNA was then used as the template for PCR amplification of *HBB* site. The PCR product was sequenced by Sanger sequencing. (D) Representative sequencing chromatographs of homozygous mutant cells (G_−28_G_−25_/G_−28_G_−25_), heterozygous cells (A_−28_G_−25_/G_−28_G_−25_), and wild-type cells (A_−28_G_−25_/A_−28_G_−25_). (E) A summary of the base editing efficiency in homozygous skin fibroblast cells from the patient. A total of 30 single cells were whole-genome amplified. And 28/30 cells were successfully amplified by PCR. Both G_−28_ and G_−25_ were converted to A (A_−28_ and A_−25_ respectively). *PCR amplification failed in 2 cells

We found 2 wild-type cells (2/28, 7.1%) with the genotype of A_−28_G_−25_/A_−28_G_−25,_ proving precise repair of both mutant alleles (Fig. 2D and 2E). Additionally, only one mutant allele (A_−28_G_−25_/G_−28_G_−25_) was repaired in 3/28 (10.7%) cells, resulting in heterozygosity (Fig. 2E). Consistent with our previous data using human cell lines (Fig. 1D), we also found G>A conversion at G_−25_ of the target site in 3/28 (10.7%) cells, highlighting the need for developing base editor variants with a narrower deamination window to improve the precision of base editing (Figs. 1D and 2E). Here, these data showed that 5/28 (17.8%) cells was repaired precisely, demonstrating the feasibility of repairing *HBB* −28 (A>G) mutation *in situ*.

### Correcting *HBB* −28 (A>G) mutation in cloned human embryos by BE3

Next, we tested the feasibility of repairing *HBB* −28 (A>G) mutation in human embryos. To model disease embryos, we generated cloned human embryos by nuclear transfer (Fig. 3A). The 1st polar body (PB1) and spindle of the *in vitro* matured oocytes were removed, and then the oocytes were fused with lymphocyte cells from peripheral blood of the patient. The reconstructed oocytes were activated and cultured until the appearance of pronucleus (PN). Approximately 5–6 h later, BE3 mRNA (200 ng/μL) and gRNA-1 (100 ng/μL) were injected into the cytoplasm after the appearance of pronucleus (Fig. S4). Of the 30 embryos injected, 26 survived (Fig. 3B). 48 h later, the *HBB* site of each embryo was PCR amplified individually. And then the PCR products were detected by Sanger sequencing and deep sequencing. *HBB* site was successfully amplified in 22/26 embryos (Fig. 3B). Interestingly, in these cloned embryos, we found high point mutation repairing efficiency, which was between 7.0% and 25.9% among the repaired embryos (Figs. 3C, S5 and Table S1). Analysis of the data showed that G_−28_ was converted to either A or C in 45.4% (10/22) of the injected embryos (Fig. 4B). In embryo #17, G_−28_ was converted to C. In the other 9 embryos, G_−28_ was converted to A, representing precise mutation repairing (Fig. 3B and 3C). Furthermore, we did not find deamination at G_−25_, indicating highly efficient and specific point mutation repairing in these embryos (Fig. 3C).

**Figure 3:**
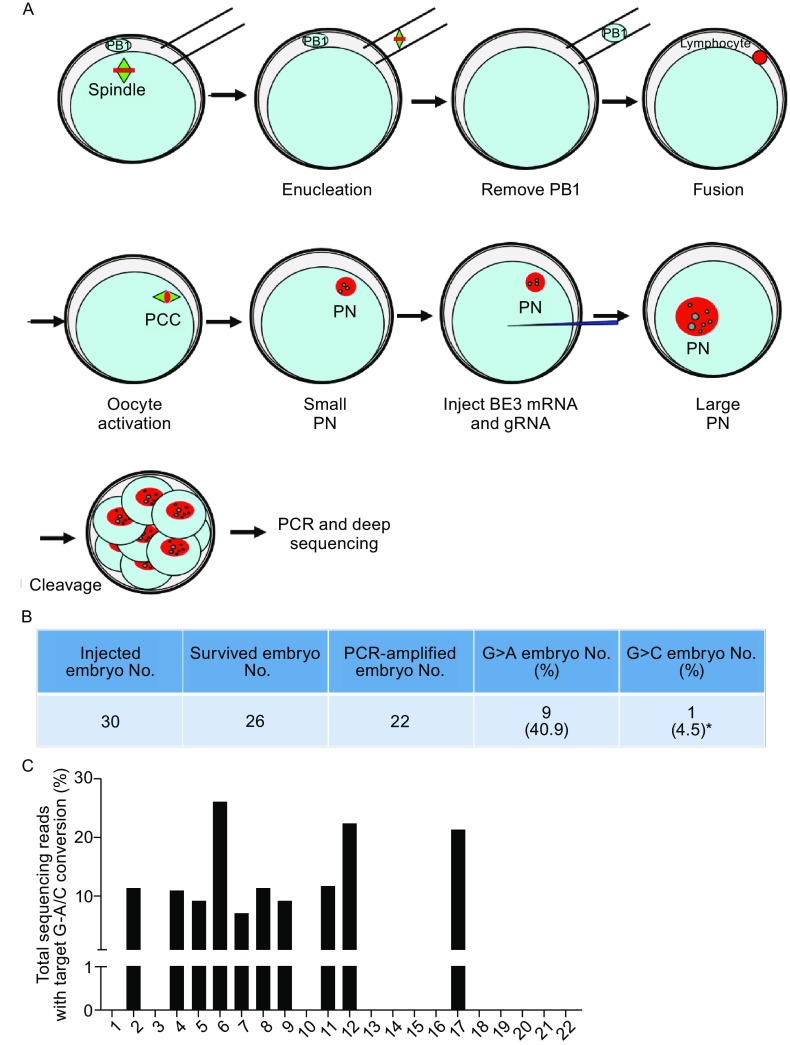
**Effective** ***HBB*** **gene correction in human embryos by BE3**. (A) Schematic of repairing *HBB* −28 (A>G) in cloned human embryos by BE3 and gRNA-1. Cloned *HBB* −28 (A>G) mutant homozygous human embryos were generated by fusing lymphocyte cell, from peripheral blood of the patient, with *in vitro* matured oocytes. And the BE3 mRNA and gRNA mixture was injected after the appearance of pronucleus. *HBB* site from each embryo was amplified by PCR and deep sequenced. PB1, the 1st polar body. PN, pronucleus. ZP, zonapellucida. (B) Summary of base editing-mediated point mutation repairing by BE3 in cloned human embryos. The repaired embryo contains G>A conversion at the *HBB* −28 site. *, The target G at the *HBB* −28 site was converted to C instead of A. (C) Deep sequencing to detect successful repairing by BE3 in human embryos. Target site PCR amplicons from these embryos were deep sequenced

**Figure 4:**
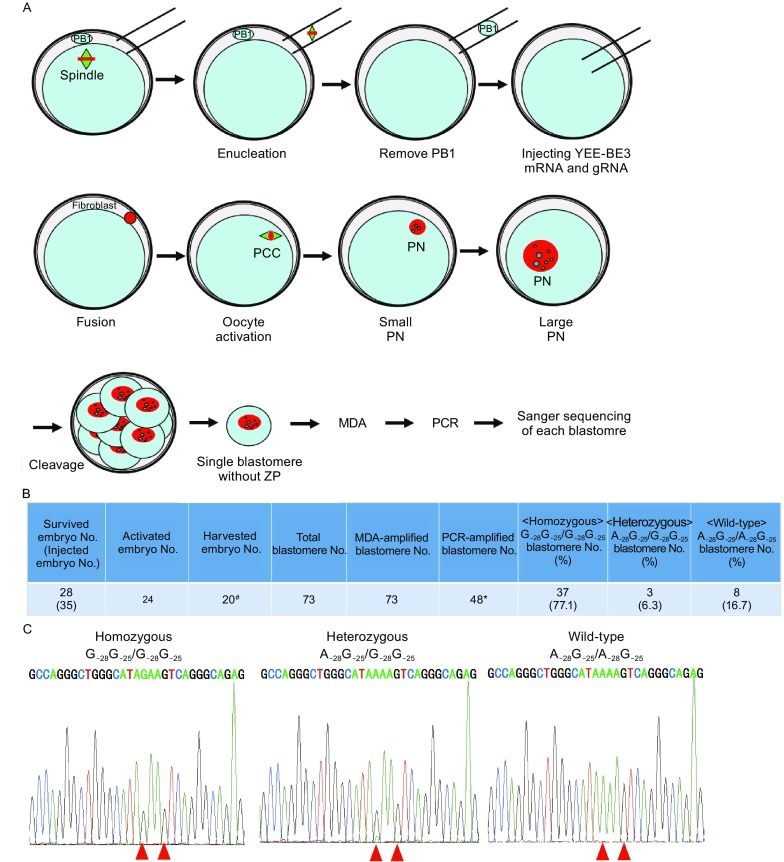
**Improving the precision of gene correction in human embryos by YEE-BE3**. (A) Schematic of repairing *HBB* −28 (A>G) in cloned human embryos by YEE-BE3 and gRNA-1. Firstly, cloned *HBB* −28 (A>G) mutant homozygous human embryos were generated by fusing skin fibroblast cell from the patient with *in vitro* matured oocytes. And YEE-BE3 mRNA and gRNA mixture was injected after removing PB1. And 1 h later, the injected oocytes were fused with skin fibroblast cells. Then the fused embryos were activated and cultured for another 48 h. Single blastomere was isolated and MDA amplified. Then *HBB* site was amplified and sequenced. PB1, the 1st polar body. PN, pronucleus. ZP, zonapellucida. (B) Summary of base editing-mediated point mutation repairing by YEE-BE3 in cloned human embryos. The numbers of homozygous mutant blastomere (G_−28_G_−25_/G_−28_G_−25_), heterozygous blastomeres (A_−28_G_−25_/G_−28_G_−25_), and wild-type blastomeres (A_−28_G_−25_/A_−28_G_−25_) were calculated. #, 4 embryos did not develop into 2-cell stage. *, *HBB* site failed to be amplified by PCR. (C) Sanger sequencing to detect successful repairing by YEE-BE3 in each blastomere. Representative sequencing chromatographs of homozygous mutant blastomeres, heterozygous blastomeres and wild-type blastomeres

### Effective *HBB* −28 (A>G) mutation repair in cloned human embryos by YEE-BE3

Although we did not find off-target deamination at G_−25_, we could not rule out the possibility of off-target deamination at G_−25_ in human embryos according to the data in human cells (Figs. 1D and 2E). We therefore turned to YEE-BE3, a BE3 variant with a smaller deamination window (Kim et al., 2017d). We injected gRNA-1 and YEE-BE3 mRNA before fusing the skin fibroblast cell with oocytes in which spindle and PB1 had been removed. Injecting YEE-BE3 mRNA before fusion will leave more time for protein translation and deamination before cell division. At about one hour after fusion, the reconstructed embryos were activated and cultured for another 48 h, when embryos were at 4–8 cell stage (Fig. 4A). The zona pellucidas of these embryos were removed, and 73 blastomeres were isolated from 20 embryos (Fig. 4B). Then the blastomeres were MDA-amplified individually (Fig. 4A). The *HBB* site was PCR amplified from these MDA products and sequenced by Sanger sequencing (Fig. 4C). We successfully amplified the *HBB* loci in 48 blastomeres (48/73, 65.8%) (Fig. 4B), and found that 37 blastomeres were still homozygous mutants (G_−28_G_−25_/G_−28_G_−25_), while the other 11 blastomeres (11/48, 22.9%) had been repaired (Fig. 4B and Table 1). A total of 3 out of 11 (6.3%) repaired blastomeres were heterozygous, and the other 8 (16.7%) were WT with both mutant alleles repaired perfectly (Table 1). More importantly, no off-target deamination at G_−25_ was observed, suggesting highly precise deamination at G_−28_.

**Table 1:** Summary of base editing-mediated point mutation repairing by YEE-BE3 in human embryos

Embryo ID	Blastomere No.	PCR-amplified blastomere No.^*^	<Homozygous> G_−28_G_−25_/G_−28_G_−25_ blastomere No. (%)	<Heterozygous> A_−28_G_−25_/G_−28_G_−25_ blastomere No. (%)	<Wild-type> A_−28_G_−25_/A_−28_G_−25_ balstomere No. (%)
#1	2	1	1	0	0
#2	5	2	2	0	0
#3	7	4	3	1 (25)	0
#4	3	1	1	0	0
#5	6	3	3	0	0
#6	6	3	2	0	1 (33.3)
#7	2	2	2	0	0
#8	1	1	1	0	0
#9	4	4	4	0	0
#10	6	3	3	0	0
#11	1	1	1	0	0
#12	4	4	3	1 (25)	0
#13	3	3	2	0	1 (33.3)
#14	2	1	0	0	1 (100)
#15	6	5	4	1 (20)	0
#16	2	1	1	0	0
#17	2	1	1	0	0
#18	5	4	1	0	3 (75)
#19	5	3	1	0	2 (66.7)
#20	1	1	1	0	0

A total of 20 embryos were harvested for single blastomere genotyping. In some blastomeres, both alleles were repaired. In one blastomere, only one mutant allele was repaired

* Some blastomeres failed to be amplified by PCR

Checking the genotype of all the blastomeres in the repaired embryos, we found that most of them were mosaic, containing homozygous mutant blastomeres and repaired blastomeres (Table 1). Of the 7 repaired embryos with more than 2 successfully sequenced blastomeres, the percentage of repaired blastomere was between 20% and 75% (Table 1). In addition, the sequenced blastomere from embryo #14, with 1 successfully sequenced blastomeres, was wild-type (1/1, 100%). The high percentage of repaired blastomere suggests the possibility of getting repaired embryos free of mosaicism. These data demonstrate that it is feasible to correct *HBB* −28 (A>G) mutation in human embryos efficiently and specifically by base editor.

## Discussion

Taken together, our data highlight the tremendous potential of correcting homozygous disease and compound heterozygous mutations by base editing in human somatic cells and embryos. Although we did not achieve 100% repair in human embryos, we and other groups have reported 100% base editing in mouse embryos (Kim et al., 2017c; Liang et al., 2017). By injecting BE3 protein and optimizing the injection time of the base editors, 100% repair of disease mutations may be achieved, as reported in CRISPR/Cas9 system (Hashimoto et al., 2016). Injecting BE3 protein may also help to improve the specificity of base editing mediated gene correction in human embryos. Moreover, we observed G>C mutation, caused by base excision repair (BER), in human cells and embryos (Komor et al., 2016). Therefore, developing new methods to inhibit base excision repair is needed, such as adding chemical inhibitors and overexpressing UGI (Wang et al., 2017). Additionally, while we found no indel formation in the cloned human embryos, indels have been observed in base editing in human cells and mouse embryos (Kim et al., 2017c; Komor et al., 2016; Liang et al., 2017). Further investigation is needed to block indel formation to improve the safety of base editing. Whether BE2 will lead to fewer indel at the *HBB* −28 sites will need further investigation.

Although we did not find off-target effects at the top 10 potential off-target sites examined, the specificity of base editors needs more comprehensive investigation through genome-wide specificity assays, such as Digenome-seq (Kim et al., 2017a). Indeed, additional genome-wide specificity assays are sorely needed for in-depth and accurate investigation of the *in vivo* specificity of base editors. Furthermore, the precision of base editors should be further improved to eliminate base conversion at G_−25_. Whether base editor variants such as YE1-BE3, YE2-BE3, EE-BE3, and YEE-BE3 will prove more appropriate warrants further investigation.

Moreover, in addition to technical issues, ethic and societal issues associated with germline gene therapy need to be investigated and discussed thoroughly before the clinical application.

Intriguingly, we found that *HBB* −28 (A>G) mutation repairing efficiency was about 20% in the constructed cell line and primary skin fibroblast cells. Although 10.7% of the repaired skin fibroblast cells were heterozygous, it is still able to cure anemia (Dever et al., 2016). Whether base editors will be equally or more efficient in human hematopoietic stem cells is still under investigation. High repairing efficiency in human hematopoietic stem cells will lead to new therapeutics for β-thalassemia intermedia and β-thalassemia major patients with *HBB* −28 (A>G) mutation.

## Materials and methods

### Ethics

This study was approved by the Ethical Committee of the First Affiliated Hospital of Sun Yat-sen University (Approval Reference Number: 2017-49). Written informed consent was obtained from each infertile couple prior to donating immature oocytes for research. Immature oocytes were donated from patients undergoing intracytoplasmic sperm injection (ICSI) from Mar 2015 to June 2017 at the Reproductive Medical Center of the First Affiliated Hospital of Sun Yat-sen University. Written informed consent was obtained from each donor prior to donating immature oocytes for researches. All of the patients followed a protocol using gonadotrophin-releasing hormone agonist and Gonal-F (Gonal-F; Merck Serono, The Netherlands) for ovarian stimulation (Ding et al., 2015). Oocyte retrieval was carried out 34–36 h after the administration of 10,000 IU HCG (Ovidrel; Merck Serono, The Netherlands). Oocytes lacking a polar body were considered immature (germinal vesicle and metaphase I oocytes) after stripping for intracytoplasmic sperm injection (ICSI) on the day of oocyte retrieval. Only the oocytes remaining at the metaphase I stage were used for *in-vitro* maturation. Written informed consent was also obtained from the β-thalassemia patients to donate blood and skin fibroblast cells for gene editing research in cells and embryos.

### Cloning of plasmids

pcDNA3.1(−)-BE3 was synthesized by Guangzhou IGE biotechnology LTD. YEE-BE3 (W90Y/R126E/R132E triple mutant) was from Addgene (#851777). The pcDNA3.1(−)-BE3 was used for expression in human cells and *in vitro* transcription. pUC19-SpCas9 gRNA expression vector was cloned by amplifying the U6-gRNA fragment from pX330 (Addgene, #42230), and then inserting this fragment into pUC19 vector. Sequences for cloning the gRNA-1, gRNA-2, and gRNA-3 into the pUC19-SpCas9 gRNA expression vector were listed in Table S2. gRNAs was cloned into pDR274 (Addgene, #42250) for *in vitro* transcription. Sequence for cloning gRNA-1 into pDR274 was listed in Table S3. Target region, spanning HBB −28 sites, was amplified using HBB-FP and HBB-RP primers (Table S4). And then the PCR product was digested with *Not*I and *Asc*I. This digested PCR product was then cloned into pENTR/D-TOPO vector (Invitrogen), resulting in pENTR/D-TOPO-HBB. −28 A>G mutation was then induced into this vector by quick change PCR using HBB-78-QC-FP and HBB-78-QC-RP primers (Table S4). And then gateway cloning was carried out to clone the −28 (A>G) mutant HBB fragment into pLenti-EF1a-DEST-SFB vector, resulting pLenti-EF1a-DEST-HBB-SFB vector.

### Generating *HBB* −28 (A>G) mutant stable cell line

pLenti-EF1a-DEST-HBB-SFB plasmids were transfected together with psPAX2 (Addgene, #12260) and pMD2.G (Addgene, #12259) into 293T cells to produce lentivirus. 48 h after transfection, the virus was harvested and used to infect 293T cells. 24 h after infection, the infected cells were selected with 1 μg/mL puromycin. After puromycin selection, 3 clones were picked and expanded. The integrated exogenous *HBB* fragment was amplified and sequenced using FP1 and RP1 (Table S4).

### Base editing in *HBB* −28 (A>G) mutant stable cell line and sequencing

*HBB* −28 (A>G) mutant stable cell line was transfected with different base editors and gRNAs. Exogenous integrated target sites and 10 potential off-target sites were amplified using primers listed in Table S5. The PCR product was used for TA cloning sequencing or deep sequencing.

### Target sites deep sequencing

Ten potential off-target sites were identified by online tool Cas-OFFinder (http://www.rgenome.net/cas-offinder/) to identify potential off-target sites. Sequences surrounding these 10 sites and integrated −28 (A>G) mutation site were PCR amplified and deep sequenced using IlluminaHiseq 2500 PE150 as paired-end 150 reads. The primers for off-target site analysis can be found in Table S3. High-throughput sequencing data was analysed as reported. Briefly, Sample sequencing was done on an IlluminaHiSeq 2000 PE150 as paired-end 150 bp reads. The merged paired-end reads of each library were separated based on barcodes in primers (Table S5) by Python scripts and then submitted to cutadapt (v1.11) for trimming primer sequence. The trimmed reads were aligned to reference sequence by means of BWA with default parameters (v0.7.13). Samtools (v1.3, http://samtools.sourceforge.net) and Picard tools (v2.2.2, http://picard.sourceforge.net) were used to build indices and sort reads. GATK (The Genome Analysis ToolKit, version 3.5) Haplotype Caller and VarScan (v2.4.2, mpileup2snp and mpileup2indel with –min-reads2 10 –min-var-freq 0.01) were used to call variants for all samples and the combined variants of which were then divided into indels and SNVs by SelectVariants. Next, we aligned the reference and repaired sequence to the reads of each barcode by bowtie (version 1.1.2, http://bowtie-bio.sourceforge.net/index.shtmL) with no mismatch. The repair rate was equal to the number of repaired reads divided by the number of reference reads.

### Base editing in skin fibroblast cells

1 × 10^5^ skin fibroblast cells was tranfected with 2 μg BE3 expression plasmid and 1 μg gRNA expression plasmid by nucleofection according to the manufacturer’s manual (Lonza, V4XP-2032).

### *In vitro* transcription

BE3 and YEE-BE3 mRNA was transcribed using the mmol/LESSAGEmmol/LACHINE T7 ULTRA kit (Life Technologies) following the manufacturer’s instruction. gRNA-1 transcribed using the MEGAshortscript T7 kit (Life Technologies) following the manufacturer’s instruction. mRNAs and gRNAs were subsequently purified using the MEGAclear kit (Life Technologies) and resuspended in RNase-free water.

### *In vitro* maturation

The *in-vitro* maturation culture medium consisted of G-IVF medium (Vitrolife Sweden AB, Goteborg, Sweden) supplemented with 10% human serum albumin (HSA) solution (Vitrolife), 25 mmol/L sodium pyruvate (Sigma), 75 IU/L recombinant FSH (Gonal-F; Merck Serono, The Netherlands), and 150 IU/L HCG (Ovidrel; Merck Serono, The Netherlands). Immature oocytes were cultured in a humidified atmosphere of 6% CO_2_, 5% O_2_, and 89% N_2_ at 37°C. The oocyte maturational status was evaluated after 15 h of *in-vitro* culture. Mature oocytes were identified if they extruded a polar body after 15 h of *in-vitro* culture and were then used for vitrification. Oocytes remaining immature after 15 h of *in-vitro* culture were considered incompetent for maturation and were discarded.

### Oocyte vitrification and warming

Oocytes *in vitro* maturation were vitrified and warmed by commercial Kitazato vitrification and warming kit according to the manufacturer’s protocol. Vitrification procedures were performed at room temperature (25–27°C). The oocytes *in vitro* maturation were transferred from the culture medium into the ES medium (KitazatoBioPharma Co, JP) for 15 min and then VS for 90 s. The oocytes were aspirated and placed on the tip of the Cryotop (KitazatoBioPharma Co, JP) and the Cryotop sheet were plunged into liquid nitrogen immediately. Warming procedures were performed by placing the Cryotop in a warming solution (TS, 1 mol/L sucrose) for 50–60 s at 37°C and moving into a dilution solution (DS, 0.5 mol/L sucrose) for 3 min at room temperature. The oocytes were transferred onto the bottom of WS1 dish with small amount of DS and kept for 5 min in WS1 solution and were then transferred onto the surface of WS2 dish with minimum amount of WS1, then kept for 5 min in WS2 on a plate warmer (37°C).

### Enucleation and fusion with donor cells

Thawed oocytes were placed into separate 10 µL manipulation droplets of G-MOPS with 5% HSA and covered with tissue culture oil. After the first polar body of the oocytes reached 12 o’clock, partial zonapellucida dissection (PZD) was performed before enucleation (Ding et al., 2015). Then, they were placed into separate 10 µL manipulation droplets of G-MOPS medium (containing 7.5 µg/mL cytochalasin B, 5% HSA) in a glass-bottom dish at 37°C for 10 min. The spindle was aspirated into the pipette with a minimal amount of cytoplasm and surrounding plasma membrane using Spindle View (Cri Inc.). Enucleated oocytes were rinsed with G-MOPS medium containing 5% HSA and incubated in G-IVF medium with 10% HSA at 37°C in 6% CO_2_, 5% O_2_, and 89% N_2_ for 60 min before fusion. PB1 was aspirated out of ZP by the pipette before enucleated oocyte fused with donor cells. Donor cells were resuspended in a drop containing HVJ-E extract (Cosmo Bio, USA) and were inserted into the perivitelline space of the enucleated oocytes. The reconstructed oocytes were kept in the manipulation medium until cell fusion was confirmed, and then the reconstructed oocytes were transferred into G-IVF medium (10% HSA) and incubated for 1 h before activation.

### Artificial activation and embryo culture

The reconstructed oocytes were parthenogenetically activated by incubation in 7.5 mol/L ionomycin (I3909, Sigma, St Louis, MO, USA) for 10 min followed by incubation in 2 mmol/L 6-dimethylamino purine (6-DMAP; d2629, Sigma, St Louis, MO, USA) for 4 h. Activated oocytes with 1 PN were injected G1 gRNA, Cas9 mRNA, and the ssDNAoligo into the cytoplasm 5–6 h after activation. The survived reconstructed embryos were cultured in microdrops of G-1 medium (Vitrolife, VitrolifeSweeden AB Göteborg, Sweeden) at 37°C in a humidified atmosphere of 6% CO_2_, 5% O_2_, and 89% N_2_ for 42 h. Blastomere of reconstructed embryos were individually aspirated out of ZP by the pipette.

### Intracytoplasmic injection of BE3 mRNA and gRNA

The mixture of BE3 mRNA (200 ng/μL) and gRNA-1 (100 ng/μL) was injected into reconstructed human embryo about 5 h after activation. And YEE-BE3 mRNA (200 ng/μL) and gRNA-1 (100 ng/μL) was injected into enucleated oocytes after the removal of PB1.

### Single embryo PCR amplification and deep sequencing

Single embryo PCR amplification was performed as described before (Zhang et al., 2016). Briefly, each embryo was transferred into a PCR tube containing 1 μL lysis buffer, and then incubated at 65°C for 3 h followed by 95°C for 10 min. The lysis product was then amplified using primers listed in Table S6.

### Whole genome amplification by multiplex displacement amplification

Whole genome amplification of the embryos was performed using the PEPLI-g Midi Kit (Qiagen). Briefly, single cell or single blastomere was transferred into PCR tubes containing reconstituted buffer D2 (7 μL), and then incubated at 65°C for 10 min, before the addition of stop solution (3.5 μL) and MDA master mix (40 μL) and incubation at 30°C for 16 h. The DNA preparation was diluted with ddH_2_O (3:100), and 1 μL of the diluted DNA was used for PCR analysis.

## Electronic supplementary material

Below is the link to the electronic supplementary material.

Supplementary material 1 (PDF 349 kb)

Supplementary material 2 (XLSX 11 kb)

Supplementary material 3 (XLSX 10 kb)

Supplementary material 4 (XLSX 10 kb)

Supplementary material 5 (XLSX 10 kb)

Supplementary material 6 (XLSX 91 kb)

Supplementary material 7 (XLSX 10 kb)
