# Supplementary material for: Correction of β-thalassemia mutant by base editor in human embryos
Source: Protein Cell. 2017 Sep 23;8(11):811–22. doi: 10.1007/s13238-017-0475-6 (PMC5676594; doi:10.1007/s13238-017-0475-6)
Supplement: Supplementary file 1 — Supplementary material 1 (PDF 349 kb) [file 13238_2017_475_MOESM1_ESM.pdf]

gRNA-1: GACTTCTATGCCCAGCCCTGG
gRNA-2: ACTTCTATGCCCAGCCCTGG
gRNA-3: CTTCTATGCCCAGCCCTGG

Figure S1. Target sequence of gRNAs targeting *HBB* -28(A>G) mutation.
The target C intended to be demethylated is in red. PAM in green.

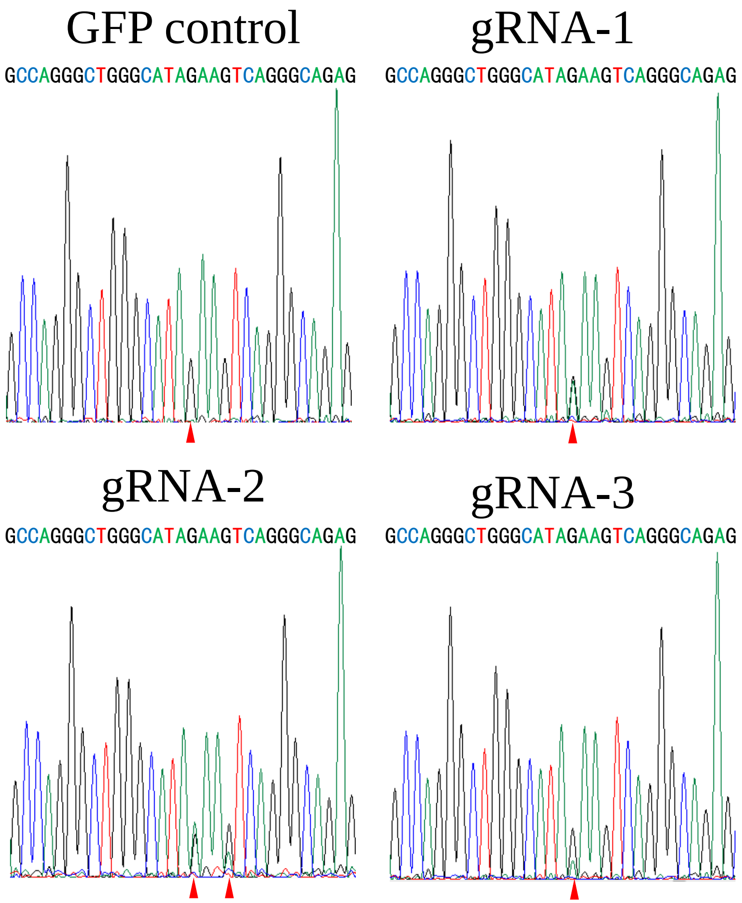

Figure S2. Precise repairing of *HBB* -28 (A>G) mutation by base editor in stable cell line detected by Sanger sequencing.

Target sites from *HBB* -28(A>G) mutant stable 293T cell lines were PCR amplified and sanger sequenced.-28(A>G) mutation and deaminated sites were labelled with red arrowhead. GFP control, *HBB* -28(A>G) mutant stable 293T cells transfected with GFP expression vector.

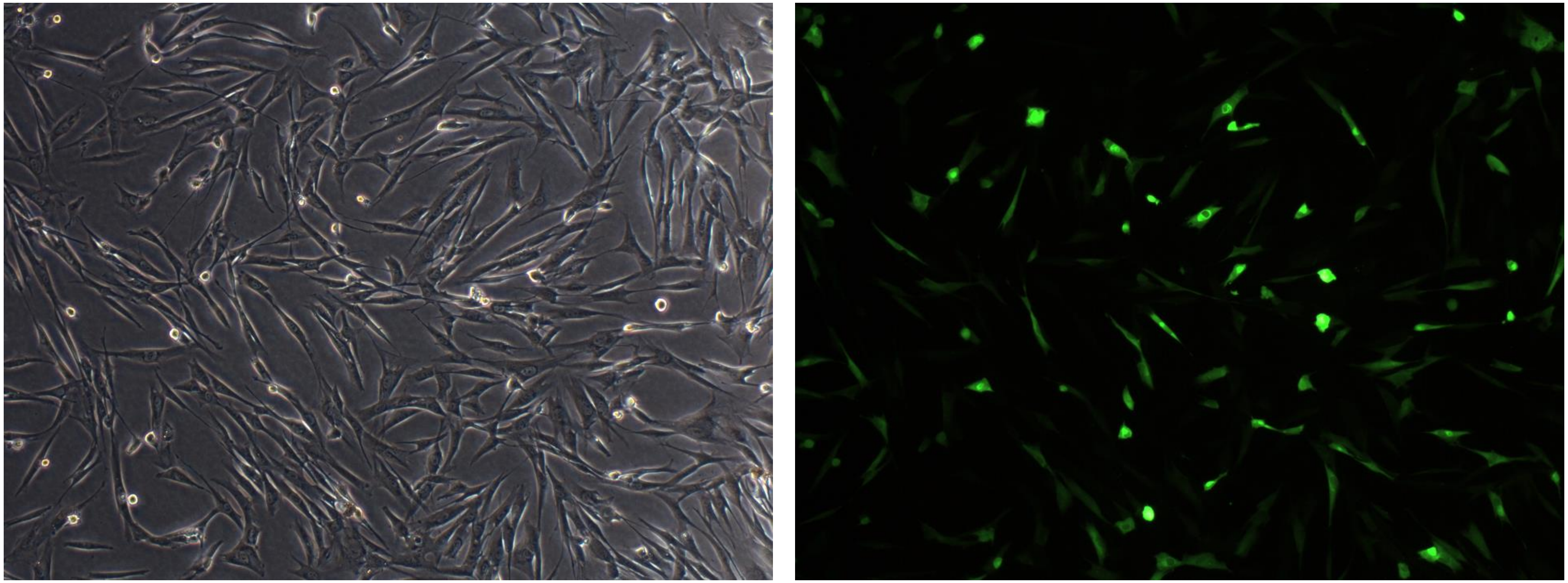

Bright Field GFP

Figure S3. Transfecting the homozygous *HBB* -28 (A>G) mutant skin fibroblast by nucleofection.

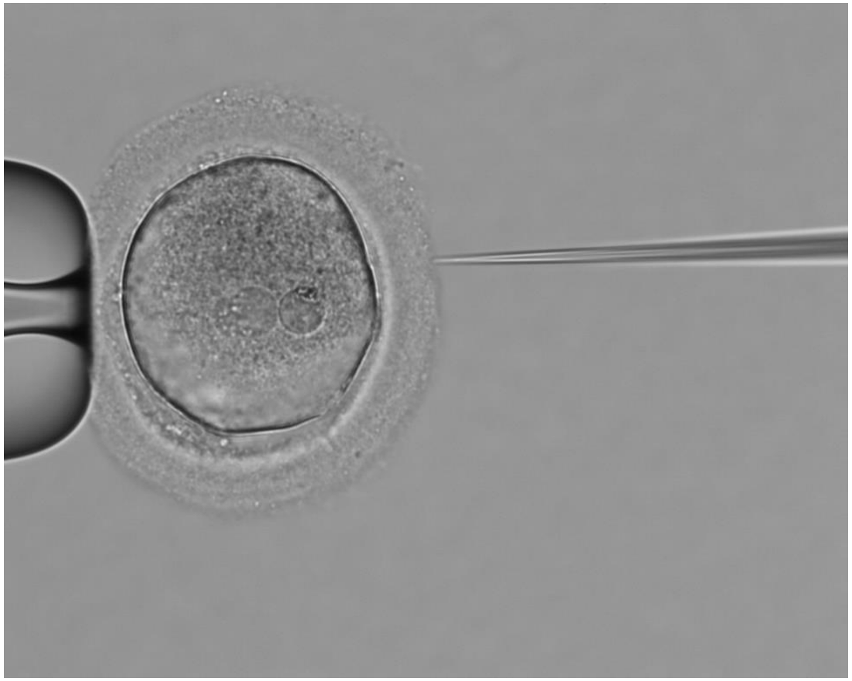

Figure S4. Injecting the cloned human embryos with gRNA-1 and BE3.

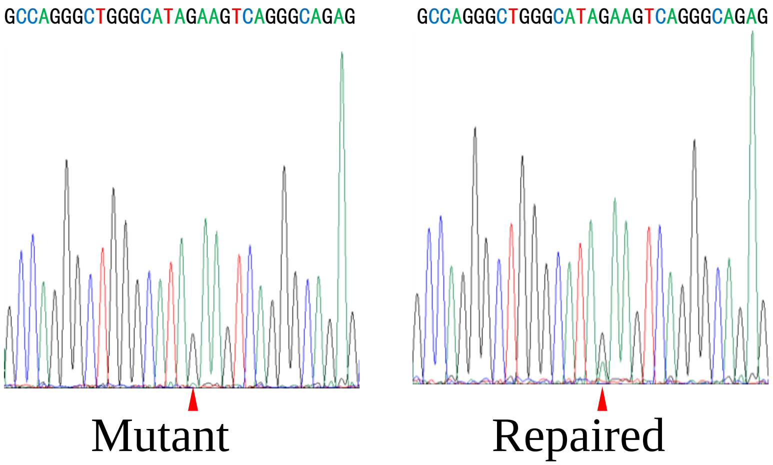

Figure S5. Sanger sequencing to detect successful repairing by base editor in cloned human embryos. Representative sequencing chromatographs of the PCR amplicons of target sites are shown here. Mutant, mutant embryo. Repaired, embryo edited by base editor. The base successfully repaired was indicated by red arrowheads.
